# Development of Keystone Macrolide Antibiotics via Selective Oxidation

**DOI:** 10.1021/acscentsci.6c00689

**Published:** 2026-05-12

**Authors:** Eléonore Moore, Amy E. Fraley

**Affiliations:** Institute of Pharmaceutical Sciences, Department of Chemistry and Applied Biosciences, ETH Zürich, Zürich 8093, Switzerland

## Abstract

A new precatalyst is developed for selective oxidation of macrolide antibiotic scaffolds.

## Graphical abstract

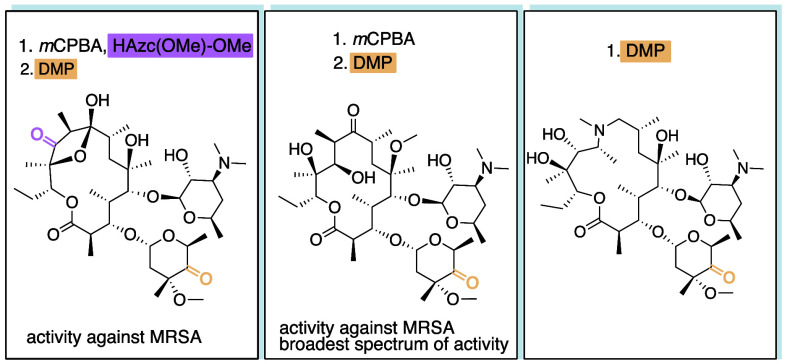

Natural products have inspired and shaped the pharmaceutical landscape through their complex and diverse molecular architectures. They often feature numerous chemical functionalities with remarkable stereo-, regio-, and chemo-selectivity, which drives their broad spectrum of activity. Natural products have, indeed, been harnessed in cancer, viral, fungal, and bacterial treatment. One such example is erythromycin A (**1**, Figure 1), and other members of the macrolide antibiotic family, which are widely prescribed and have had a significant impact on the pharmaceutical industry. They promote cell death by binding to the 50S ribosomal subunit, thereby blocking the translation machinery.^1^ However, the constant emergence of resistant bacterial strains necessitates efficient and rapid access to novel macrolide analogues. In a recent issue of *ACS Central Science*, Scott J. Miller and co-workers^2^ tackled this challenge by developing a new aminoxyl-based precatalyst (**2**, Figure 1) for site-divergent oxidation of macrolide antibiotics. In their natural form, these scaffolds contain multiple secondary alcohols with comparable reactivity, making them difficult to target without side reactions and product degradation. However, their efficient oxidation to non-native carbonyl groups could provide chemical handles for further functionalization and expand their bioactivity against resistant strains. To achieve this, Scott J. Miller and co-workers designed and synthesized a more sterically accessible azaadamantyl oxoammonium precatalyst (HAzc­(OMe)-OMe, **2**).

**Figure 1:**
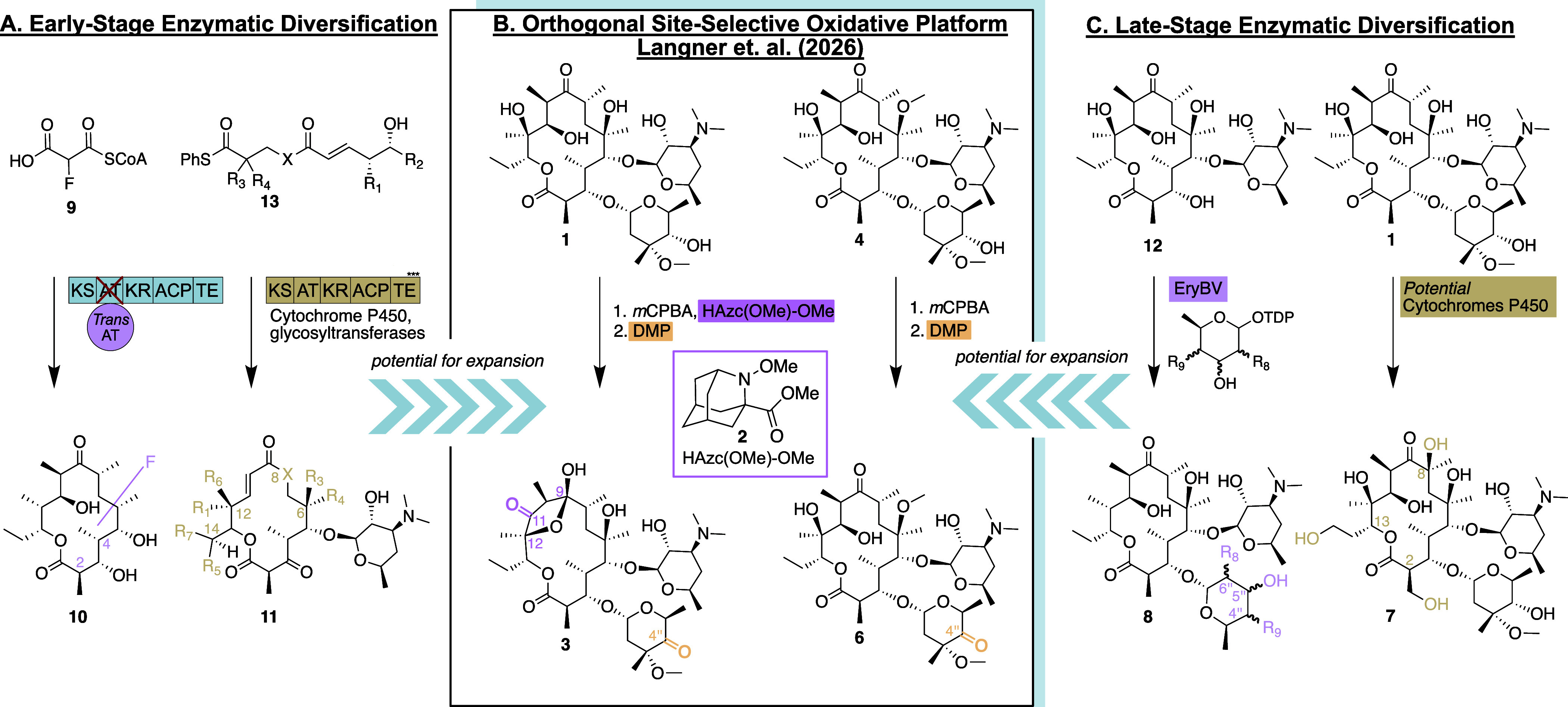
(A) Potential for early-stage enzymatic diversification by polyketide synthase engineering. R_1_, R_3_, R_4_, or R_5_ = H or Me, R_2_ = H, Me, or Et, R_6_, R_7_ = H or OH, X = CHMe, NH, or O. (B) Site-selective oxidation of antibiotic scaffolds via the HAzc­(OMe)-OMe catalyst developed in the work presented here from Scott J. Miller et al.^2^ (C) Potential for late-stage enzymatic diversification by tailoring enzyme engineering. The pictured cytochrome P450 catalyzed reactions have not yet been realized but are based on oxidations observed on other polyketide scaffolds. R_8_ = H or OH, R_9_ = OH or NHMe. KS, Ketosynthase; AT, Acyltransferase; KR, Ketoreductase; ACP, Acyl Carrier Protein; TE, Thioesterase; *m*CPBA, *meta*-chloroperoxybenzoic acid; DMP, Dess–Martin Periodinane.

The researchers also established an orthogonal macrolide-dependent oxidative catalytic platform. Remarkably, their catalyst enabled the highly selective oxidation of the C11 hydroxyl group in erythromycin A (**1**). This transformation disrupted the hydrogen bond between the C11 hydroxyl and C9 ketone, shifting the equilibrium toward the formation of the C9–C12 hemiketal. This molecule subsequently served as the basis for the synthesis of diverse analogues using orthogonal oxidants (**3**, Figure 1). Notably, this strategy led to the discovery of new analogues with only minor structural changes that were active against bacterial strains resistant to staple antibiotics erythromycin A, clarithromycin, and azithromycin.

Notably, this strategy led to the discovery of new analogues with only minor structural changes that were active against bacterial strains resistant to staple antibiotics erythromycin A, clarithromycin, and azithromycin.

Surprisingly, when the methodologies for modifying erythromycin A (**1**) were applied to related substrates such as clarithromycin (**4**, Figure 1) and azithromycin (**5**), the HAzc­(OMe)-OMe (**2**) catalyst displayed markedly different catalytic activity. This divergence is striking given the minimal structural variation; for instance, erythromycin A (**1**) and clarithromycin (**4**) differ only at C6-OMe. This subtle change led to a loss of oxidative activity, with no hydroxyl oxidation detected. However, after treatment with *meta-*chloroperoxybenzoic acid (*m*CPBA) followed by Dess–Martin periodinane (DMP), the C4″ alcohol moiety was oxidized with high selectivity (**6**, Figure 1). This is remarkable, as the same treatment applied to erythromycin A (**1**) yielded a complex mixture with overoxidation of the substrate. Azithromycin (**5**) required only DMP to access the C4″ keto-derivative. Notably, for three ostensibly similar macrolide structures, three distinct oxidative routes had to be devised. Moreover, the authors surmise that the alcohol oxidation arises from a mechanism dependent on the formation of a covalent N–O bond between the hydroxyl group and oxoammonium nitrogen. By comparing the computed density functional theory (DFT) reaction pathways of erythromycin A (**1**), clarithromycin (**4**), and azithromycin (**5**), or relevant fragments thereof, future work could decipher the differential reaction barriers for the integral steps, such as attack of the C11-OH on the oxoammonium nitrogen and the Cope-type elimination to release the carbonyl product. This would ultimately shed light on the intricacies behind the distinct reactivity of this catalyst on superficially similar substrates. A greater understanding of these complex interactions could facilitate the expansion of this catalytic system’s utility.

This work demonstrated the practicality of late-stage functionalization (site-selective oxidation) for rapid diversification and improvement of macrolide antibiotics.

Given that this work demonstrated the practicality of late-stage functionalization (site-selective oxidation) for rapid diversification and improvement of macrolide antibiotics, we envision that these methods could be applied in a combinatorial fashion with state-of-the-art biocatalytic or synthetic biology platforms developed in the last decade for similar scaffolds. The approaches could utilize native oxidizing biocatalysts, the cytochrome P450 enzymes (Figure 1C) that naturally oxidize C12, C14, and C16 scaffolds.^3,4^ The use of cytochrome P450 monooxygenases for site-selective C–H hydroxylation could be paired with the new oxoammonium-mediated alcohol oxidations to generate higher-order oxidation cascades without competing reactivity (e.g., **7** in Figure 1C). Moreover, using enzymes, the sugar moiety (Figure 1C) could be engineered to obtain novel glycosylation patterns with existing or unnatural motifs, which could impact the site-selectivity of the oxoammonium and broaden the glycosylation pattern (e.g., **8** in Figure 1C). Indeed, studies have shown that by modifying the sugar moiety, activity against erythromycin A-resistant bacteria could be recovered,^5^ which is congruent with the findings of the paper by Scott J. Miller and co-workers.^2^ This is particularly relevant to the current work because one of the analogues, C4″-keto-clarithromycin (**6**), has the broadest activity spectrum and, more importantly, activity against methicillin-resistant *Staphylococcus aureus* (MRSA).

In addition to late-stage modification options, these scaffolds could also be manipulated by early-stage incorporation of modified precursors (Figure 1A) for the diversification of the final antibiotic macrolides. Pioneering studies demonstrated that fluorinated erythromycin analogues could be produced via incorporation of fluorinated extender units (e.g., **9** in Figure 1A) into engineered polyketide synthases (PKSs) (**10** in Figure 1A).^6,7^ The thioesterase domain of the PKS could be engineered via directed evolution to facilitate the acceptance of non-native intermediates (indicated by TE*** in Figure 1A).^8^ This evolution-guided approach was coupled with late-stage oxidation and glycosylation to successfully generate numerous 14-membered macrolactones in a chemoenzymatic fashion.^9^ One could envision feeding these new-to-nature macrolactones into the oxidation pipeline developed in the work highlighted here.

The foundational work conducted in this study provides an exceptional platform for oxidative expansion of polyketide space. From a synthetic chemistry standpoint, these results deepen our understanding of the intricate interplay between the oxidant (HAzc­(OMe)-OMe, *m*CPBA, DMP) and molecular architecture of the polyketide scaffold, thereby enabling the rational selection of the best route to access the desired antimicrobial product. This work revealed novel macrolide antibiotics via a late-stage functionalization approach, and with the integration of nature’s catalytic toolbox via biocatalysis or synthetic biology, has the potential to amplify efforts to combat the rise of resistant pathogens.
